# A Compact Operational Amplifier with Load-Insensitive Stability Compensation for High-Precision Transducer Interface

**DOI:** 10.3390/s18020393

**Published:** 2018-01-29

**Authors:** Zhanghao Yu, Xi Yang, SungWon Chung

**Affiliations:** 1 Department of Electrical Engineering, University of Southern California, Los Angeles, CA 90089, USA; sungwon@ieee.org; 2 Department of Electrical Engineering and Computer Science, Massachusetts Institute of Technology, Cambridge, MA 02139, USA; xiyang@mit.edu

**Keywords:** analog integrated circuits, operational amplifiers, transducer interface circuit, Internet of Things (IoT) device

## Abstract

High-resolution electronic interface circuits for transducers with nonlinear capacitive impedance need an operational amplifier, which is stable for a wide range of load capacitance. Such operational amplifier in a conventional design requires a large area for compensation capacitors, increasing costs and limiting applications. In order to address this problem, we present a gain-boosted two-stage operational amplifier, whose frequency response compensation capacitor size is insensitive to the load capacitance and also orders of magnitude smaller compared to the conventional Miller-compensation capacitor that often dominates chip area. By exploiting pole-zero cancellation between a gain-boosting stage and the main amplifier stage, the compensation capacitor of the proposed operational amplifier becomes less dependent of load capacitance, so that it can also operate with a wide range of load capacitance. A prototype operational amplifier designed in 0.13-μm complementary metal–oxide–semiconductor (CMOS) with a 400-fF compensation capacitor occupies 900-μm${}^{2}$ chip area and achieves 0.022–2.78-MHz unity gain bandwidth and over 65${}^{\circ }$ phase margin with a load capacitance of 0.1–15 nF. The prototype amplifier consumes 7.6 μW from a single 1.0-V supply. For a given compensation capacitor size and a chip area, the prototype design demonstrates the best reported performance trade-off on unity gain bandwidth, maximum stable load capacitance, and power consumption.

## 1. Introduction

The internet of things (IoT) is a new paradigm, which connects any physical objects embedded with ambient computational intelligence to each other such that these objects can recognize others and exchange collected data [1,2,3]. With the advent of the Internet of Things, there has been an increasing demand on the transducers that are able to perform diverse functions such as sensors, actuators, and radio frequency identification tags, for a wide variety of applications including communication, imaging, display, finance, data centers, transportation, health-care, and biomedical devices [4,5,6,7,8,9,10].

Capacitive micromachined ultrasonic transducers (CMUTs) [11,12,13], piezoelectric transducers [14,15,16], and electro-neural stimulators [17,18,19,20,21], in particular, need to drive nonlinear capacitive load with a large impedance variation. With such a variable capacitive load, in order to achieve an extremely high-resolution control (e.g., 16-bit resolution) to the extent well beyond the present state-of-the-art, which is typically implemented with less than 6–8-bit resolution [22,23,24], the electronic interface circuitry of such transducers requires a precision high-gain operational amplifier. However, it is challenging to design an internally compensated high-gain operational amplifier particularly when the amplifier needs to drive a very wide-range of load capacitance but an available chip area for the amplifier is limited [25,26,27,28,29,30,31,32,33,34,35,36,37,38,39,40,41,42,43].

A sufficient direct-current (DC) voltage gain can be generated by multi-stage amplifiers [32,33,34,35,36,37,38,39,40,41]. However, multi-stage operational amplifiers suffer from stability problems with variable capacitive loads. Although frequency compensation techniques are commonly used in multi-stage amplifiers to improve feedback stability [25,26,27,28,29,30,31,32,33,34,35,36,37,38,39,40,41], these conventional compensation techniques do not allow a wide range of load capacitance. In addition, a compensation capacitor occupies a large chip area, especially when a high capacitive load exists. The pseudo single-stage (PSS) amplifier [42], which in fact is a multi-stage amplifier, was recently introduced to improve the feedback stability by decreasing its first-stage gain to reduce the compensation capacitor size.

In this paper, we present a gain-boosted two-stage operational amplifier, whose compensation capacitance size is less sensitive of load capacitance compared to the previously reported operational amplifiers. Compared to the PSS amplifier, rather than decreasing the first-stage gain [42], the proposed amplifier alternating-current-couples the first-stage and adds a gain-boosting stage to the second-stage input, which provides a higher flexibility in frequency compensation and also allows low-power operation and a higher unity-gain frequency. The compensation capacitor size of a prototype operational amplifier is up to four orders of magnitude smaller than the load capacitance. By combing the conventional Miller compensation with a pole-zero cancellation technique, compared to the previously reported high-gain operational amplifiers, the proposed operational amplifier allows the smallest compensation capacitor size for a given load capacitance [38,39,40,41,42,43].

The remainder of this paper is organized as follows. Section 2 reviews the previously known operational amplifier stability compensation techniques. Section 3 presents the operation and architectural analysis of the proposed operational amplifier with a novel stability compensation technique. The detailed circuit implementation and pole-zero cancellation analysis are given in Section 4. Simulation results and performance comparison with the present state-of-the-art are presented in Section 5. Concluding remarks are stated in Section 6.

## 2. Review on Stability Compensation Topologies

### 2.1. Conventional Miller Compensation

**Figure 1:**
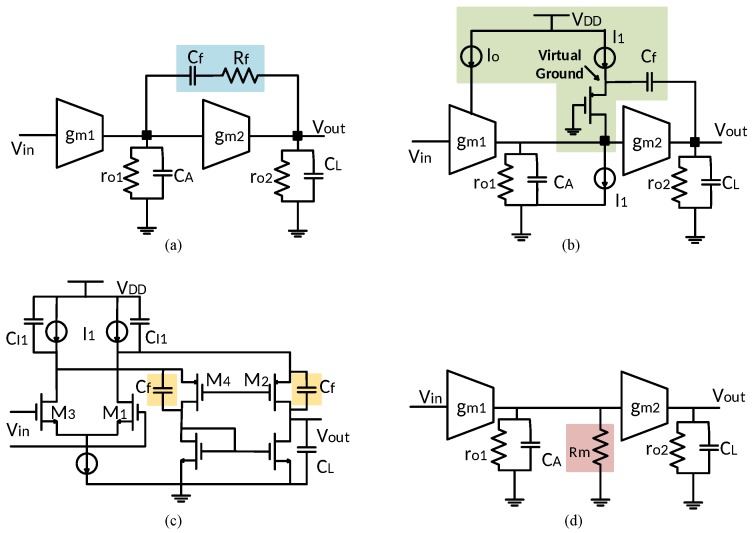
(**a**) Conventional Miller frequency compensation [25]. (**b**) Ahuja frequency compensation [26]. (**c**) Conventional feedforward frequency compensation [27]. (**d**) Pseudo single-stage amplifier [42]. (Diagrams were redrawn with simplification in order to facilitate the comparison among different compensation techniques.)

Miller compensation, which has been extensively used in integrated operational amplifiers, deals with stability issues in frequency response by introducing capacitor $C_{f}$ in series with resistance $R_{f}$ between the input and output stage. Figure 1a illustrates the architecture of a two-stage amplifier using conventional Miller compensation [25,44]. Its transfer function from the input $V_{in}$ to the output $V_{out}$ is

(1) $$\begin{array}{cc} \frac{V_{\mathrm{out}}\left(s\right)}{V_{\mathrm{in}}\left(s\right)}= & \;g_{m1}\;r_{o1}\;g_{m2}\;r_{o2}\times \frac{\left(1+\frac{S}{\omega_{z}}\right)}{\left(1+\frac{S}{\omega_{p1}}\right)\left(1+\frac{S}{\omega_{p2}}\right)}, \end{array}$$

where the dominant pole $\omega_{p1}$, second pole $\omega_{p2}$ and zero $\omega_{z}$ are given as

(2) $$\begin{array}{cc} \omega_{z}= & \;-\frac{g_{m2}}{C_{f}\;\left(1-g_{m2}R_{f}\right)}, \end{array}$$

(3) $$\begin{array}{cc} \omega_{p1}= & \;\frac{1}{r_{o1}C_{f}\;\left(1+g_{m2}r_{o2}\right)}, \end{array}$$

(4) $$\begin{array}{cc} \omega_{p2}= & \;\frac{g_{m2}C_{f}}{C_{A}C_{L}+C_{A}C_{f}+C_{L}C_{f}}, \end{array}$$

the pole splitting that the output pole is transfered to the second pole is achieved by the Miller compensation [25,44]. The zero can be moved to left half plane by increasing the compensating resistance value $R_{f}$. Assuming that intrinsic capacitance $C_{A}$ is much smaller than load capacitance $C_{L}$ and compensation capacitance $C_{f}$, the unity gain frequency $\omega_{u}$ is $g_{m1}/C_{f}$, results in

(5) $$\begin{array}{cc} \frac{\omega_{p2}}{\omega_{u}}= & \;\frac{g_{m2}\;C_{f}}{g_{m1}\;C_{L}}. \end{array}$$

To avoid the unity gain frequency higher than $\omega_{p2}$, which may cause stability issues, the compensating capacitance must be designed larger than $C_{L}g_{m1}/g_{m2}$. Therefore, the conventional Miller compensation topology meets tremendous challenges in driving large capacitive load with a limited footprint.

### 2.2. Ahuja Compensation

An improved compensation technique was introduced by Ahuja. It utilizes the current transformer providing virtual ground to eliminate feed-forward path in Miller compensation [26], as shown in Figure 1b. The dominant pole $\omega_{p1}$ is lightly changed from (3) to

(6) $$\begin{array}{cc} \omega_{p1}= & \;\frac{1}{r_{o1}C_{f}\;g_{m2}r_{o1}}, \end{array}$$

while the second pole $\omega_{p2}$ now is

(7) $$\begin{array}{cc} \omega_{p2}= & \;\frac{g_{m2}\;C_{f}}{C_{A}\left(C_{L}+C_{f}\right)}, \end{array}$$

which is higher than (4) [27]. The unity gain frequency $\omega_{u}$ is still given by $g_{m1}/C_{f}$. As a result, the ratio between the second pole $\omega_{p2}$ and unity gain frequency $\omega_{u}$ is augmented to

(8) $$\begin{array}{cc} \frac{\omega_{p2}}{\omega_{u}}= & \;\frac{g_{m2}}{g_{m1}}\;\frac{C_{f}}{C_{A}}\;\frac{C_{f}}{C_{f}+C_{L}}. \end{array}$$

Compared to the conventional Miller compensation, a smaller compensating capacitance is required to drive a same load capacitance for a given phase margin. It copes better with heavy capacitive load [26,45]. However, this reduction is still heavily restricted by the capacitive load.

### 2.3. Conventional Feedforward Compensation

The feedforward compensation technique is introduced to obtain high-frequency performance by implementing pole-zero cancellation [46,47]. Its principle in a folded-cascode amplifier is illustrated in Figure 1c. Assuming poles are widely spread, the positions of zeros and poles are approximately given by

(9) $$\begin{array}{cc} \omega_{z}= & \;\frac{g_{m2}}{\left(C_{f}+C_{\mathrm{BD}2}\right)}, \end{array}$$

(10) $$\begin{array}{cc} \omega_{p1}= & \;\frac{1}{r_{o}\;C_{1}}, \end{array}$$

(11) $$\begin{array}{cc} \omega_{p2}= & \;\frac{g_{m2}}{\left[C_{2}+C_{3}+C_{f}+\frac{\left(C_{f}+C_{\mathrm{BD}2}\right)\left(C_{2}+C_{\mathrm{BD}1}\right)}{C_{1}}\right]}, \end{array}$$

where $C_{1}=C_{L}+C_{\mathrm{GD}2}$, $C_{2}=C_{I1}+C_{\mathrm{GS}1}$, $C_{2}=C_{\mathrm{BD}1}+C_{\mathrm{BD}2}$, and the $r_{o}$ is the output impedance of this amplifier [27]. For the amplifier without $C_{f}$, the zero is much higher than the second pole as $C_{\mathrm{BD}2}$ is normally much smaller than the capacitors of the second pole. By inserting the feedforward capacitance $C_{f}$, the zero $\omega_{z}$ shifts to lower frequency and is practical to cancel the second pole $\omega_{p2}$. However, due to the parasitic capacitances, mismatch between the zero and the second pole always exists. It should be noted that the second pole also shifts to lower position, because of $C_{f}$, even though with a minor degree. Consequently, a relatively large $C_{f}$ is needed to alleviate this mismatch. This limitation can be relieved by adding a resistance $R_{f}$ in series with $C_{f}$ [27].

### 2.4. Pseudo Single-Stage Amplifier

The single-stage amplifier only have one high-impedance node at its output, that is why it can drive a large load capacitance without any stability issues. For the pseudo single-stage amplifier, an introduced intermediate resistance $R_{m}$ is paralleled with first-stage output impedance $r_{o1}$, as shown in Figure 1d, it significantly reduces the output impedance of first stage. Its dominant pole $\omega_{p1}$ and second pole $\omega_{p2}$ are obtained as

(12) $$\begin{array}{cc} \omega_{p1}= & \;\frac{1}{r_{o2}\;C_{L}}, \end{array}$$

(13) $$\begin{array}{cc} \omega_{p2}= & \;\frac{1}{(r_{o1}\left|\right|R_{m})\;C_{A}}, \end{array}$$

because of the small $R_{m}$, the second pole is much higher than the dominant pole [42]. This two-stage amplifier has a frequency response similar to a single-stage amplifier without compensation capacitance. However, this topology is implemented at the expense of insufficient DC voltage gain. This problem can be alleviated by adding a gain booster to the schematic.

In summary, there are mainly two problems existing in these previous frequency compensation topologies. First, the size of compensation capacitor is tightly limited by the load capacitance. Specifically, for a 1-nF load capacitance, the compensation capacitance needed to address stability issues is conventionally larger than 10 pF, which occupies a large proportion of the chip area. In addition, these techniques are required to be optimized based on the single specific load capacitance. As a result, the wide range of load capacitance cannot be driven. In next section, a novel two-stage operational amplifier is introduced to solve these two problems by combing Miller compensation and pole-zero cancellation.

## 3. Proposed Architecture

**Figure 2:**
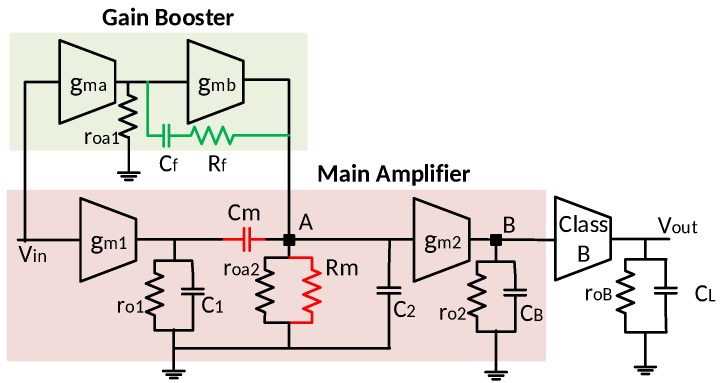
Architecture of the proposed gain-boosted two-stage operational amplifier with load-insensitive stability compensation.

The proposed operational amplifier architecture is illustrated in Figure 2. It consists of a main amplifier, a gain booster, and a Class-B output stage.

In the main amplifier, the first stage transconductance $g_{m1}$ is alternating-current (AC) coupled to the second stage transconductance $g_{m2}$ through the capacitor $C_{m}$, the voltage gain at point A is generated by the gain booster. The transfer function from the input $V_{in}$ to the point A $V_{A}$ is

(14) $$\begin{array}{cc} \frac{V_{A}\left(s\right)}{V_{\mathrm{in}}\left(s\right)}= & \;g_{\mathrm{ma}}\;\left[r_{\mathrm{oa}1}\left|\right|\frac{\left(R_{f}+\frac{1}{s\;C_{f}}\right)}{1+g_{\mathrm{mb}}(r_{\mathrm{oa}2}\left|\right|r_{o1}\left|\right|R_{m})}\right]\times g_{\mathrm{mb}}\;\left[R_{m}\left|\right|r_{\mathrm{oa}2}\left|\right|r_{o1}\left|\right|\frac{1}{s\;(C_{2}+C_{1}||C_{m})}\right], \end{array}$$

where $g_{\mathrm{ma}}$ and $g_{\mathrm{mb}}$ are the transconductance, $r_{\mathrm{oa}1}$ and $r_{\mathrm{oa}2}$ are the output impedance, of the first and second stage of gain booster, respectively. $r_{o1}$ is the output impedance of the first stage, and $R_{m}$ is the inter-stage load impedance, in main amplifier. Because of the Miller effect, the impedance of $C_{f}$ in series with $R_{f}$ is amplified by $[1+g_{\mathrm{mb}}(r_{\mathrm{oa}2}\left|\right|R_{m})]$, where $g_{\mathrm{mb}}(r_{\mathrm{oa}2}\left|\right|R_{m})$ is the DC voltage gain of the second stage of gain booster. The voltage gain between A and B is

(15) $$\begin{array}{cc} \frac{V_{B}\left(s\right)}{V_{A}\left(s\right)}= & \;g_{m2}\;\left(r_{o2}\left|\right|\frac{1}{s\;C_{B}}\right), \end{array}$$

where $r_{o2}$ is the output impedance of the second stage of main amplifier. Therefore, the overall transfer function from the input $V_{in}$ to the output $V_{out}$ is obtained as

(16) $$\begin{array}{cc} \frac{V_{\mathrm{out}}\left(s\right)}{V_{\mathrm{in}}\left(s\right)} & =\frac{V_{A}\left(s\right)}{V_{\mathrm{in}}\left(s\right)}\times \frac{V_{B}\left(s\right)}{V_{A}\left(s\right)}\times \frac{V_{\mathrm{out}}\left(s\right)}{V_{B}\left(s\right)}\\ & =\;g_{\mathrm{ma}}\;\left[r_{\mathrm{oa}1}\left|\right|\frac{\left(R_{f}+\frac{1}{s\;C_{f}}\right)}{1+g_{\mathrm{mb}}(r_{\mathrm{oa}2}\left|\right|r_{o1}\left|\right|R_{m})}\right]\times g_{\mathrm{mb}}\;\left[R_{m}\left|\right|r_{\mathrm{oa}2}\left|\right|r_{o1}\left|\right|\frac{1}{s\;(C_{2}+C_{1}||C_{m})}\right]\;\\ & \;\times g_{m2}\;\left(r_{o2}\left|\right|\frac{1}{s\;C_{B}}\right)\;g_{\mathrm{mB}}\left(r_{\mathrm{oB}}\left|\right|\frac{1}{s\;C_{L}}\right), \end{array}$$

where $g_{\mathrm{mB}}$ and $r_{\mathrm{oB}}$ is the transconductance and output impedance of the Class-B output stage, respectively. Compared to other capacitors in this function, $C_{B}$ is much smaller and negligible. This function is approximated to

(17) $$\begin{array}{cc} \frac{V_{\mathrm{out}}\left(s\right)}{V_{\mathrm{in}}\left(s\right)}≃ & \;g_{\mathrm{ma}}\;r_{\mathrm{oa}1}\;g_{\mathrm{mb}}\;(r_{\mathrm{oa}2}\left|\right|R_{m})\;g_{m2}\;r_{o2}\;g_{\mathrm{mB}}\;r_{\mathrm{oB}}\times \frac{\left(1+\frac{S}{\omega_{\mathrm{zg}}}\right)}{\left(1+\frac{S}{\omega_{\mathrm{pg}}}\right)\;\left(1+\frac{S}{\omega_{p1}}\right)\;\left(1+\frac{S}{\omega_{p2}}\right)}, \end{array}$$

where

(18) $$\begin{array}{cc} \omega_{\mathrm{zg}}= & \frac{1}{C_{f}\;R_{f}}, \end{array}$$

(19) $$\begin{array}{cc} \omega_{\mathrm{pg}}= & \frac{1}{r_{\mathrm{oa}1}\;C_{f}\;g_{\mathrm{mb}}(r_{\mathrm{oa}2}\left|\right|R_{m})}, \end{array}$$

(20) $$\begin{array}{cc} \omega_{p1}= & \frac{1}{r_{\mathrm{oB}}C_{L}}, \end{array}$$

(21) $$\begin{array}{cc} \omega_{p2}= & \frac{1}{(R_{m}\left|\right|r_{o1}\left|\right|r_{\mathrm{oa}2})\left(C_{1}\left|\right|C_{m}+C_{2}\right)}. \end{array}$$

Note that $\omega_{\mathrm{pg}}$ is the dominant pole introduced by the gain booster, and $\omega_{\mathrm{zg}}$ is the zero of the gain booster. $\omega_{p1}$ is the amplifier output node pole, and $\omega_{p2}$ is the pole from node A, and $g_{\mathrm{mB}}$ is the transconductance of the Class-B output stage.

**Figure 3:**
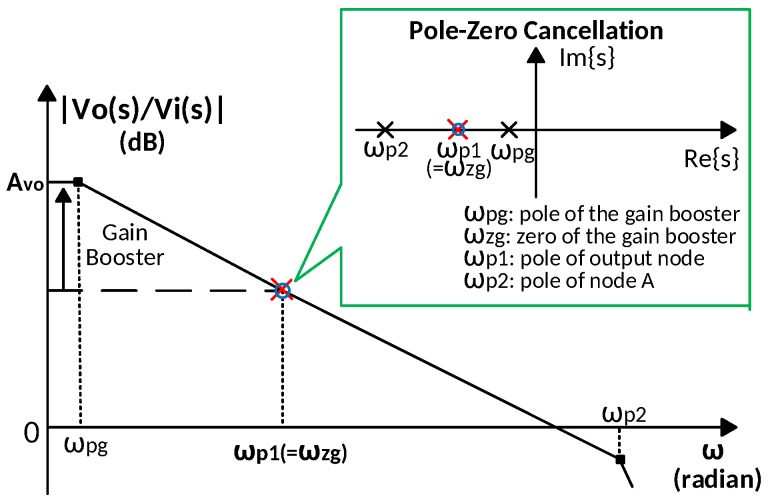
The gain booster provides a dominant pole $\omega_{\mathrm{pg}}$ and the zero of the gain booster $\omega_{\mathrm{zg}}$ cancels the main amplifier output node pole $\omega_{p1}$, thereby making the second pole $\omega_{p2}$ independent of the load capacitance $C_{L}$.

Figure 3 illustrates the frequency response of the proposed amplifier. The DC gain is boosted by $g_{\mathrm{ma}}r_{\mathrm{oa}1}$. The gain booster not only increases the DC gain of the main amplifier, but also moves the dominant pole from $\omega_{p1}$ to $\omega_{\mathrm{pg}}$, which is independent of $C_{L}$, as shown in Equation (19). However, the extra pole from the gain booster output node reduces the overall phase margin. The Miller compensation by $C_{f}$ and $R_{f}$ in the gain booster alleviates this problem. The zero of the gain booster $\omega_{\mathrm{zg}}$ is designed to cancel the output node pole, $\omega_{p1}$, by choosing a suitable $C_{f}$ and $R_{f}$. The pole-zero cancellation enable the proposed work to drive a wide range of load capacitance. The compensation capacitor size comparison between the pseudo single-stage (PSS) amplifier amplifier and this work is given in Table 1. Compared to a PSS amplifier, by taking advantage of Miller compensation, the proposed amplifier reduces the compensation capacitor size by ten times while providing the same DC gain and unity gain bandwidth (UGBW).

**Figure 4:**
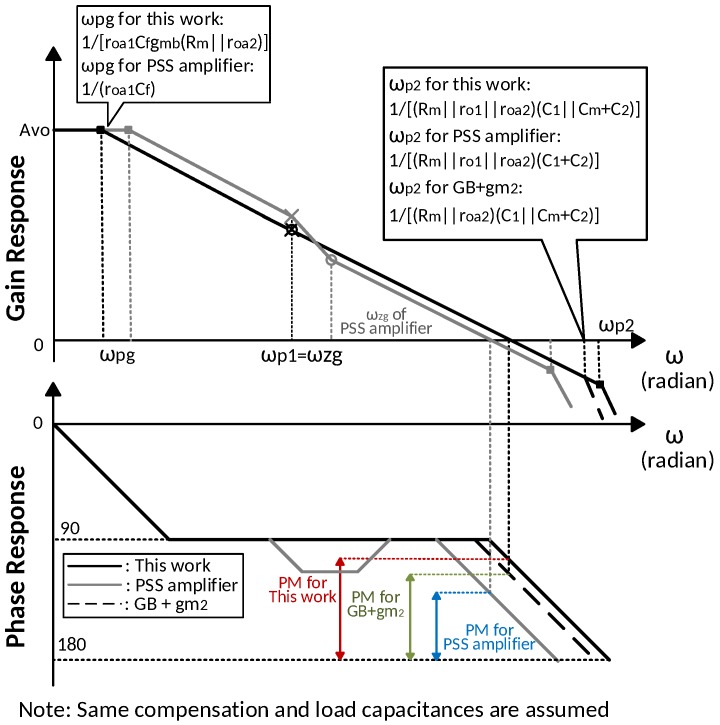
Frequency response for this work, the PSS amplifier [42] and the design without the first stage of main amplifier (GB+ $g_{m2}$).

The stability of the operational amplifier is dominated by its phase margin, which is the difference between the phase and 180${}^{\circ }$ at the unity-gain cut-off frequency. For a two-pole system, assuming poles are widely spread, its phase margin (PM) is given as

(22) $$\begin{array}{cc} \mathrm{PM} & =180{}^{\circ }-\frac{180{}^{\circ }}{\pi }\tan^{-1}\left(\frac{\omega_{u}}{\omega_{p1}}\right)-\frac{180{}^{\circ }}{\pi }\tan^{-1}\left(\frac{\omega_{u}}{\omega_{p2}}\right)\\ & ≃\;90{}^{\circ }-\frac{180{}^{\circ }}{\pi }\tan^{-1}\left(\frac{\omega_{u}}{\omega_{p2}}\right), \end{array}$$

where the $\omega_{p1}$ and the $\omega_{p2}$ are the dominant and second pole respectively, the $\omega_{u}$ is the unity gain frequency. For the proposed work, the phase margin is primarily determined by the pole from the node A, $\omega_{p2}$, as shown in (21), which is not affected by the load capacitance $C_{L}$. The capacitance at node A is much smaller than the compensation capacitance and load capacitance. The gain booster and $R_{m}$ raise the second pole location from $1/(C_{A}r_{o1})$ to $1/[C_{A}(r_{o1}\left|\right|r_{\mathrm{oa}2}\left|\right|R_{m})]$ where $C_{A}$ is approximately but less than $\left(C_{1}+C_{2}\right)$ and $C_{m}$ is designed to be much larger than $C_{1}$ and $C_{2}$. Then the ratio between the second pole and the unity gain frequency is increased, which broadens the phase margin. Compared to the design without $g_{m1}$, the proposed amplifier moves $\omega_{p2}$ higher from $1/(C_{A}(r_{\mathrm{oa}2}\left|\right|R_{m}))$ to $1/[C_{A}(r_{o1}\left|\right|r_{\mathrm{oa}2}\left|\right|R_{m})]$. The comparison about frequency response bode plots among this work, the PSS amplifier [42] and the design without $g_{m1}$ is shown in Figure 4. With same compensation and load capacitance, this work achieves a larger phase margin and wider unity gain bandwidth.

While the two-stage amplifier implementing conventional Miller compensation has a second pole determined by the load capacitance, as shown in Equation (4), the dominant and second pole of this work. which are shown in Equations (19) and (21), are both independent of the load capacitance. As a result, this work can maintain a sufficient phase margin with various load capacitance, and this frequency compensation topology is less sensitive to the load capacitance compared to the conventional Miller compensation.

## 4. Circuit Implementation and Analysis

### 4.1. Circuit Implementation

**Figure 5:**
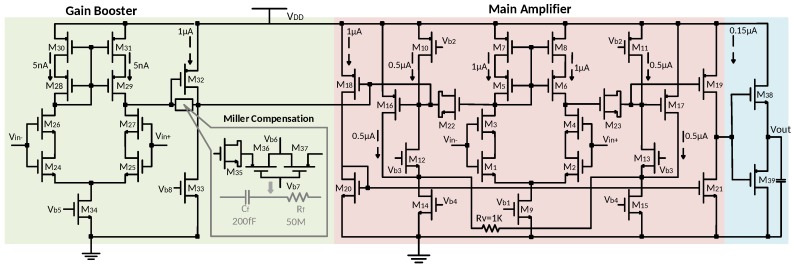
Schematic of the proposed gain-boosted two-stage operational amplifier with load-insensitive stability compensation.

Figure 5 shows the circuit of the proposed operational amplifier. Its transistor size is shown in Table 2. The composite cascode [48] is used as input stage in both the main amplifier and the gain booster in order to obtain sufficient DC voltage gain. Compared to the single transistor with doubled length, the composite pair can provide higher gain. A previous work indicated that the voltage gain exceeding 80 dB per stage can be achieved by the composite cascode configuration with transistors operating in weak or moderate inversion domain [49]. A Class-B output stage is implemented to provide a fast settling time by improving the amplifier slew rate.

**Figure A1:**
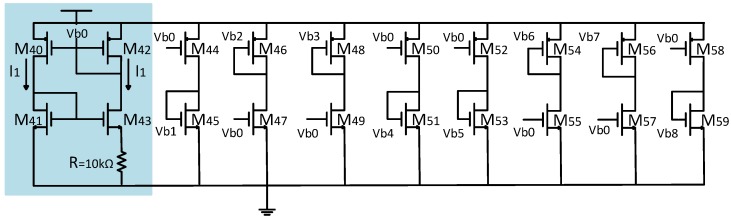
Bias circuit for the proposed work.

Design parameters of the proposed amplifier are shown in Table 3. The inter-stage load resistance $R_{m}$ can be realized by a negative feedback loop as

(23) $$\begin{array}{c} R_{m}=\frac{1}{g_{\mathrm{mv}}}+\frac{2}{g_{\mathrm{mf}}\;R_{v}\;g_{\mathrm{mv}}}, \end{array}$$

where the $g_{\mathrm{mv}}$ is the transconductance of $M_{16}$ and $M_{17}$, and the $g_{\mathrm{mf}}$ is the transconductance of $M_{12}$ and $M_{13}$ [42]. The $R_{m}$ is optimized though choosing suitable $g_{\mathrm{mv}}$ and $g_{\mathrm{mf}}$ to make the second pole $\omega_{p2}$ much higher than unity gain frequency to obtain sufficient phase margin. $R_{f}$ is replaced by floating tunable CMOS resistors to reduce the chip area [50]. The bias circuit for this work is illustrated in Figure A1 and Table A1.

### 4.2. Pole-Zero Cancellation and Sensitivity Analysis

**Figure 6:**
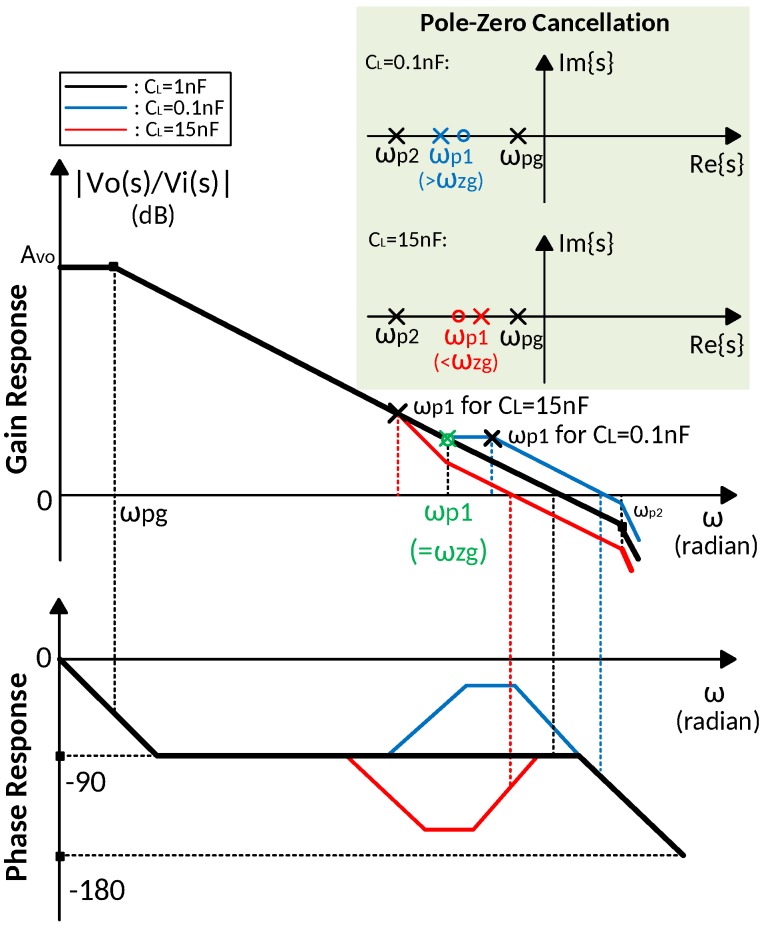
Bode plots of pole-zero cancellation with a 0.1–15-nF load capacitance, precise pole-zero cancellation occurs at $C_{L}$ = 1 nF.

In order to drive a wide range of load capacitance, $\omega_{\mathrm{zg}}$ is designed to cancel $\omega_{p1}$ with a load capacitance in-between 0.1 nF and 15 nF, as shown in Figure 6. the $\omega_{\mathrm{zg}}$ is designed lower than the output node pole $\omega_{p1}$ with minimum load capacitance, which is 0.1 nF. With the increase of load capacitance, the $\omega_{p1}$ gets lower and the precise pole-zero cancellation occurs. Then as the load capacitance further increases, the $\omega_{p1}$ get smaller than the $\omega_{\mathrm{zg}}$, and the precise pole-zero cancellation condition is broken, as a result, the phase margin become worse. To prevent a large degradation in the amplifier phase margin, the zero is designed to cancel the $\omega_{p1}$ with 1-nF load capacitance. Since a precise pole-zero cancellation is difficult to realize, especially when the load capacitance has a large variation, the pole-zero doublet of $\omega_{p1}$ and $\omega_{\mathrm{zg}}$ may degrade the settling time.

**Figure 7:**
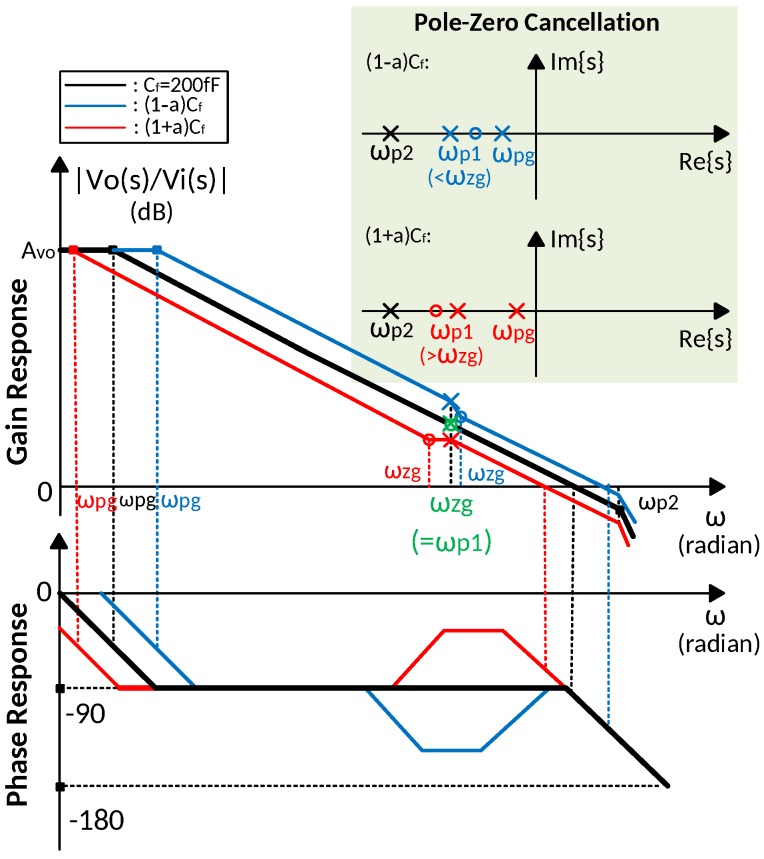
Bode plots of pole-zero cancellation over variations on Miller compensation capacitance for 1–nF load capacitance.

The effect of Miller compensation capacitance variation on the precise pole-zero cancellation is illustrated in Figure 7. The variation on $C_{f}$ causes a change on both zero and pole introduced by the gain booster, then the unity gain frequency is also changed, while the second pole is fixed. For a +10% change, both $\omega_{\mathrm{zg}}$ and $\omega_{\mathrm{bg}}$ is decreased by 10%, while the ratio between second pole and unity gain frequency is enlarged result in wider phase margin. In contrast, for a −10% variation, both $\omega_{\mathrm{zg}}$ and $\omega_{\mathrm{bg}}$ in increased by 10%, and phase margin is degenerated. The change on the unity gain frequency is approximately obtained as

(24) $$\begin{array}{cc} \Delta \omega_{u}≃ & \;\frac{1}{1+a}\left[\frac{1}{C_{f}\;R_{f}}-\frac{1}{r_{\mathrm{oa}1}\;C_{f}\;g_{\mathrm{mb}}(r_{\mathrm{oa}2}\left|\right|R_{m})}\right], \end{array}$$

where a is the variation on $C_{f}$. Because the unity gain frequency $\omega_{u}$ is much higher than $\omega_{p1}$, which is $1/(C_{f}R_{f})$, compared to $\omega_{u}$, $\Delta \omega_{u}$ is negligible. Consequently, the pole-zero cancellation is insensitive to the variation on Miller compensation capacitance.

### 4.3. Common-Mode Rejection Ratio Analysis

The common-mode rejection ratio (CMRR) of the differential amplifier reflects its ability of rejecting identical signal components on both inputs. The CMRR is defined as

(25) $$\begin{array}{cc} \mathrm{CMRR}= & \;\frac{|A_{\mathrm{DM}}|}{|A_{\mathrm{CM}}|}, \end{array}$$

where $A_{\mathrm{DM}}$ is the differential-mode gain and $A_{\mathrm{CM}}$ is the common-mode gain. It determines the attenuation applied to the noise from environment. The high CMRR is crucial for the instruments which usually work in noisy environment.

For the proposed amplifier, its common-mode rejection ratio at low frequency is typically determined by the first stage of the gain booster. Assuming that the intrinsic gain of the transistor $g_{m}r_{o}$ is much larger than one, the differential-mode gain of this stage is approximately obtained as

(26) $$A_{\mathrm{DM},\mathrm{gb}1}≃\;g_{m25}\;(g_{m27}r_{o27}r_{o25}\;||\;g_{m29}r_{o29}r_{o31}),$$

while its common-mode gain is approximated as

(27) $$\begin{array}{c} A_{\mathrm{CM},\mathrm{gb}1}≃\;\frac{1}{2g_{m31}r_{o34}}, \end{array}$$

Then the CMRR of the proposed work at DC is

(28) $$\begin{array}{cc} \mathrm{CMRR} & =\frac{\left|A_{\mathrm{DM},\mathrm{gb}1}\right|}{|A_{\mathrm{CM},\mathrm{gb}1}|}\\ & =\;\frac{1}{2g_{m31}r_{o34}}\times \;g_{m25}(g_{m27}r_{o27}r_{o25}\;||\;g_{m29}r_{o29}r_{o31})\\ & =104\mathrm{dB}, \end{array}$$

which is close to the simulated CMRR of 109 dB.

## 5. Simulation Results and Discussion

**Figure 8:**
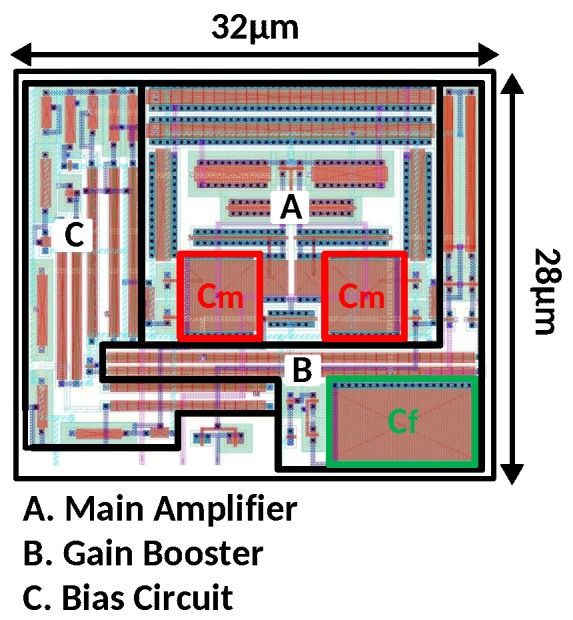
Layout of the proposed amplifier in 130-nm technology.

The proposed amplifier is designed using a 130-nm CMOS technology with a total area of 0.00090 ${\mathrm{mm}}^{2}$. Its layout is shown in Figure 8. The proposed amplifier without bias circuit occupies 0.00073-${\mathrm{mm}}^{2}$ chip area. The total stability compensation area is 100 μm${}^{2}$.

**Figure 9:**
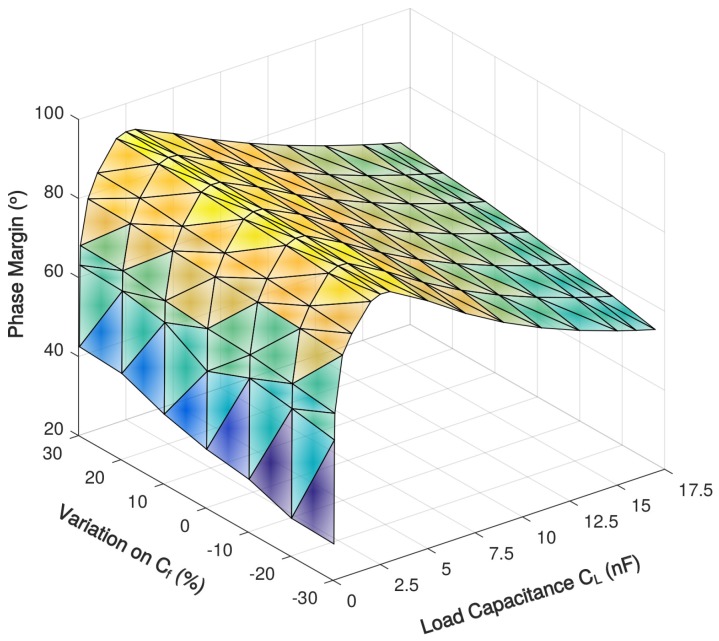
Simulated phase margin of the proposed amplifier with 0.05–17 nF load capacitance over Miller compensation capacitance variations.

**Figure 10:**
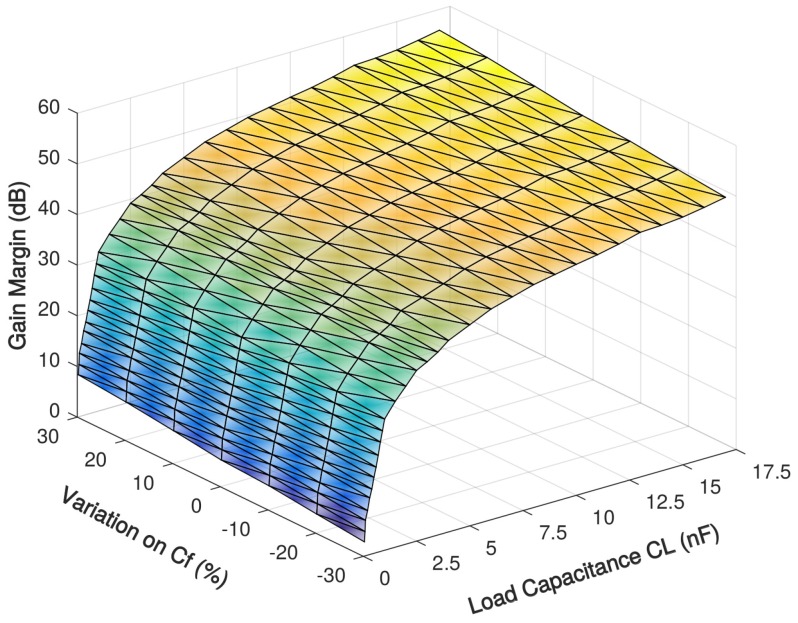
Simulated gain margin of the proposed amplifier with 0.05–17 nF load capacitance over Miller compensation capacitance variations.

The stability simulation with 0.05–17 nF load capacitance over Miller compensation capacitances variations are shown in Figure 9 and Figure 10 and Table 4 and Table 5. For the prototype amplifier, the maximum phase margin is achieved when load capacitance $C_{L}$ is 2.5 nF, which is 91${}^{\circ }$. The phase margin larger than 70${}^{\circ }$ with a load capacitance of 0.2–12 nF and it becomes less than 65${}^{\circ }$ when the load capacitance exceeds the 0.1–15-nF range. With the 30% variation on Miller compensation capacitance, the largest change in phase margin is less than 8% and demonstrates a good tolerance on process variation.

**Figure 11:**
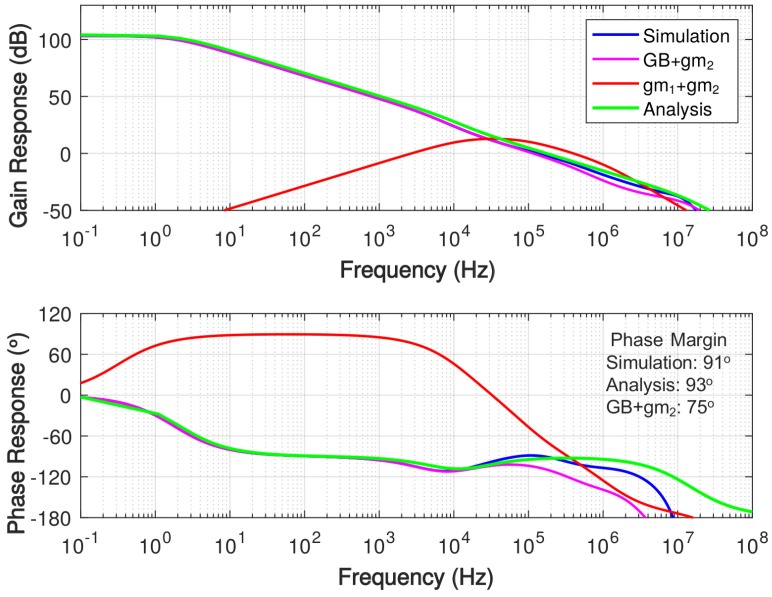
Simulated frequency response of the proposed amplifier with 2.5-nF load capacitance.

**Figure 12:**
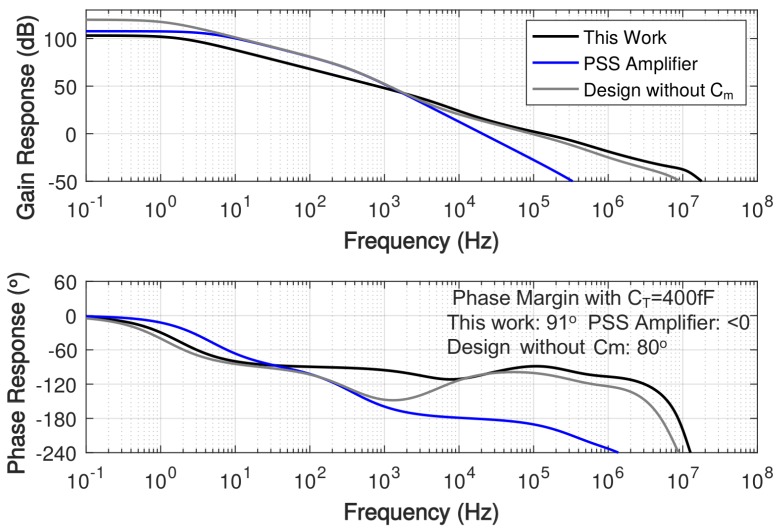
Comparison of simulated frequency response among this work, the PSS amplifier [42] and the design without $C_{m}$ with 2.5-nF load capacitance and 400-fF total compensation capacitance.

The simulated frequency response with a 2.5-nF load capacitance is shown in Figure 11, demonstrating 130-kHz unity-gain frequency and over 100-dB gain at DC. The frequency response of the amplifier without the gain booster is separately simulated, which is labeled as $g_{m1}+g_{m2}$, showing a significant DC gain drop as expected from the AC coupling between $g_{m1}$ and $g_{m2}$. In addition, the frequency response of the amplifier without the first stage of the main amplifier ($g_{m1}$) is also simulated, which is labeled as GB + $g_{m2}$, showing 16${}^{\circ }$ degradation in the phase margin. It also shows that the first stage transconductance allows the overall amplifier to provide a sufficient gain at the frequencies higher than 50 kHz. Figure 12 shows the comparison of simulated frequency response among this work, the pseudo single-stage (PSS) amplifier and the design without $C_{m}$ using total compensation capacitance of 400-fF. Because the 400-fF compensation capacitance is far from sufficient for the PSS amplifier to implement pole-zero cancellation, its phase margin is degenerated severely.

**Figure 13:**
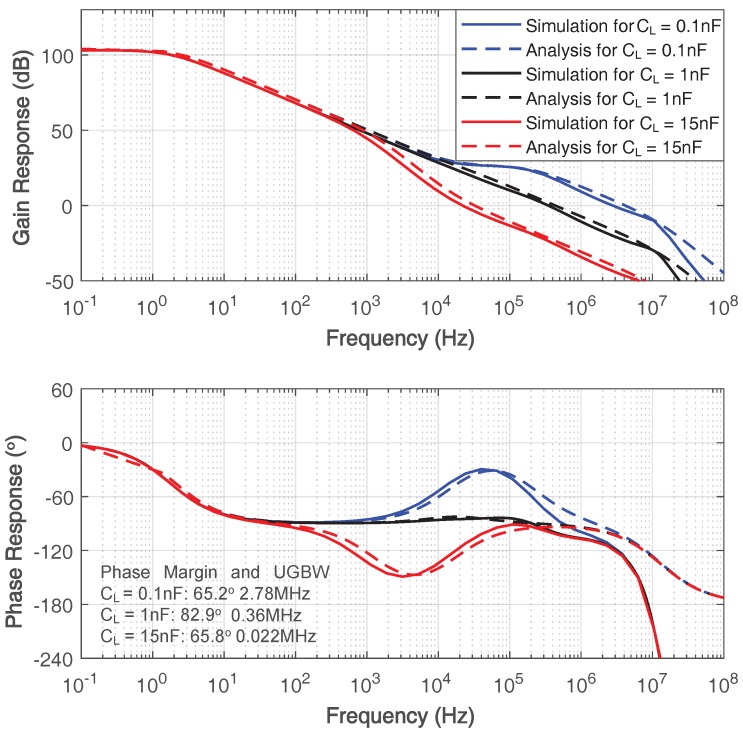
Simulated frequency response of the proposed amplifier with 0.1-nF, 1-nF and 15-nF load capacitance.

Figure 13 illustrates the simulated frequency response of the proposed amplifier with 0.1-nF, 1-nF and 15-nF load capacitances, respectively. The precise pole-zero cancellation occurs when load capacitance is 1 nF. The frequency response for 0.1-nF load capacitance shows that the pole of the gain booster, $\omega_{\mathrm{gz}}$ is lower than the output node pole $\omega_{p1}$, while $\omega_{\mathrm{gz}}$ is higher than $\omega_{p1}$ when load capacitance is 15 nF. The broken pole-zero cancellation reflected in Figure 13 accords with the analysis in previous section.

**Figure 14:**
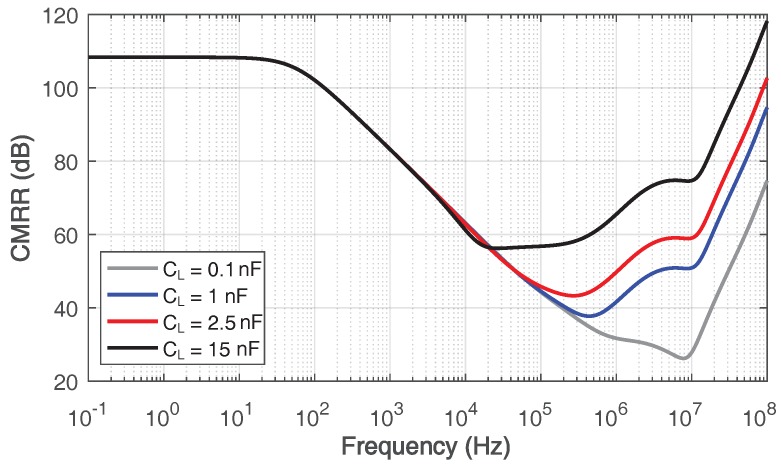
Simulated common-mode rejection ratio of the proposed amplifier with 0.1–15-nF load capacitance.

The simulated common-mode rejection ratio (CMRR) is given in Figure 14, which is 109 dB at DC. At low frequency, the high CMRR is mainly contributed by the first stage of the gain booster, which is a differential composite cascode amplifier, since the first and second stage of the main amplifier are AC coupled by $C_{m}$. With the increase of frequency, the first stage of the main amplifier begins to make a difference to the CMRR. Owing to this, the proposed work obtains a sufficient CMRR at high frequency.

**Figure 15:**
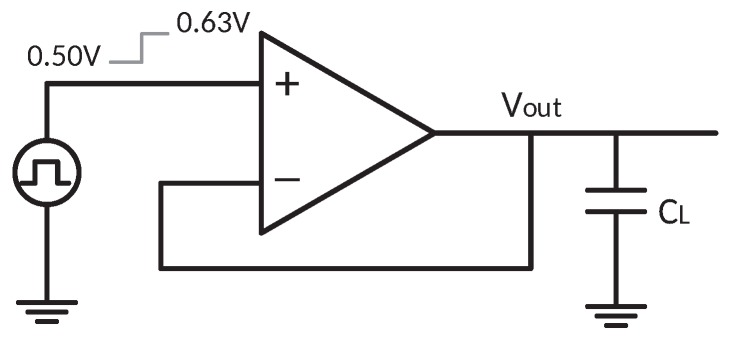
Test-bench circuit for step response simulation.

**Figure 16:**
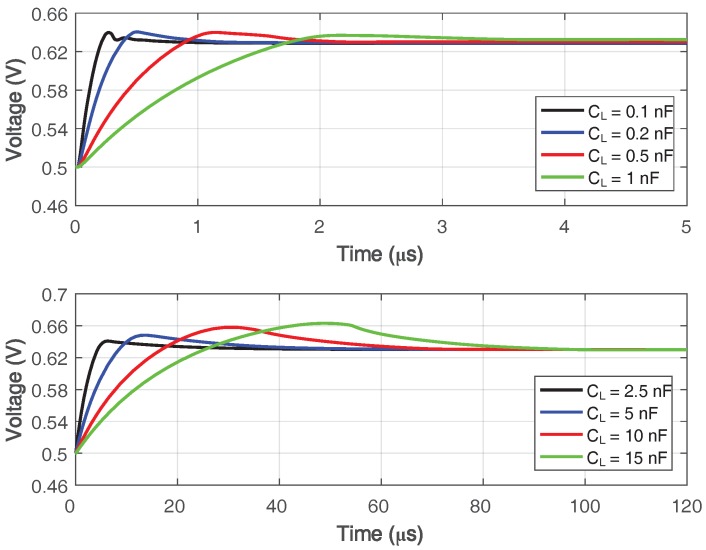
Simulated step response of the proposed amplifier with various load capacitance.

**Figure 17:**
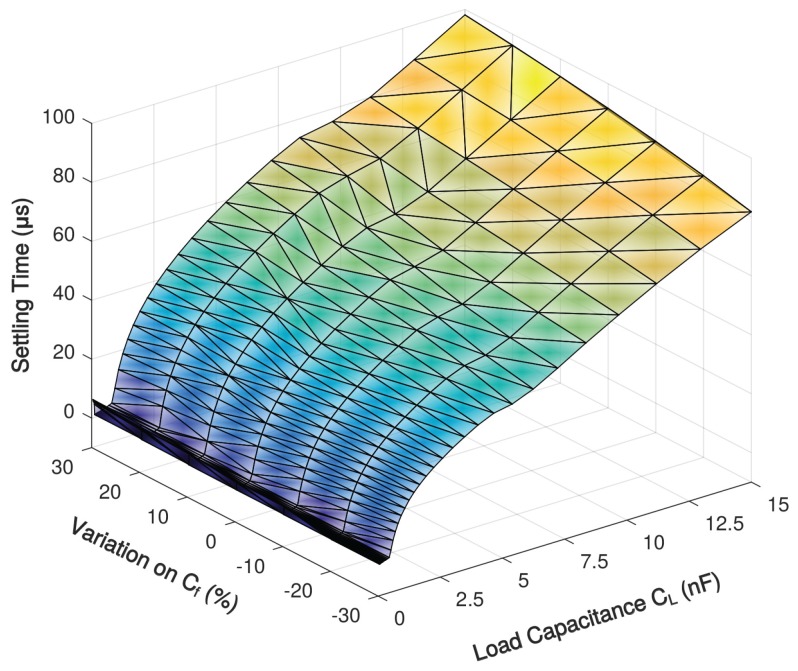
Simulated 1% settling time of the proposed amplifier with 0.1–15-nF load capacitance over Miller compensation capacitor variations.

Under a transient simulation setup illustrated in Figure 15, a step response simulation with various load capacitance is shown in Figure 16, and the simulated 1% settling time with variation on Miller compensation capacitor $C_{f}$ is shown in Figure 17. The 1% settling time is damaged due to exceeding-80${}^{\circ }$ phase margin when driving 1–7-nF load capacitance. With the diminution of $C_{f}$, the phase margin decreases, then the 1% settling time is improved.

**Figure 18:**
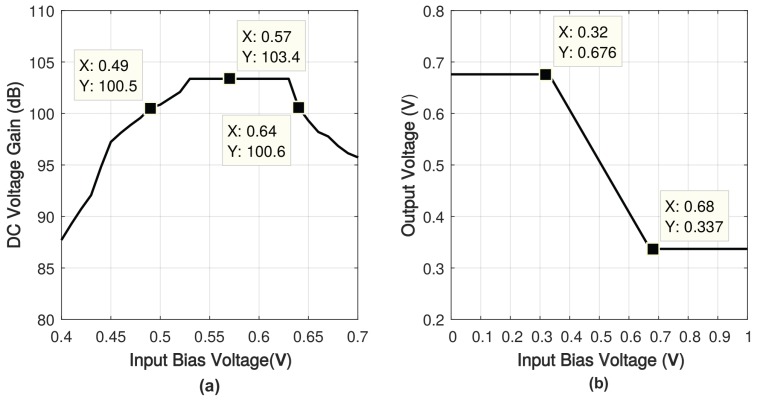
Simulated (**a**) input common-mode range (**b**) output swing of the proposed amplifier.

**Figure 19:**
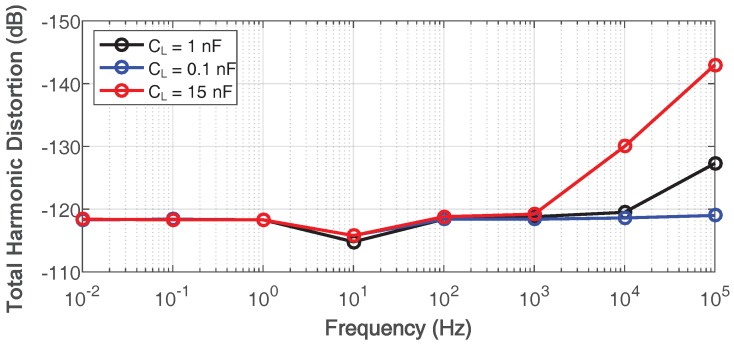
Simulated total harmonic distortion of the proposed amplifier with 0.1-nF, 1-nF and 15-nF load capacitance.

The input common-mode range (ICMR) and output swing of the proposed amplifier are shown in Figure 18. The input common range is from 0.49 V to 0.64 V, and the output bias voltage swings between 0.34 V and 0.68 V. Considering that the Class-B output stage may cause the distortion of output signal, the simulated total harmonic distortion (THD) with 40-mVpp differential input when load capacitance is 0.1 nF, 1 nF and 15 nF is shown in Figure 19. The largest total harmonic distortion is −114 dB, while the DC voltage gain is 103 dB.

**Figure 20:**
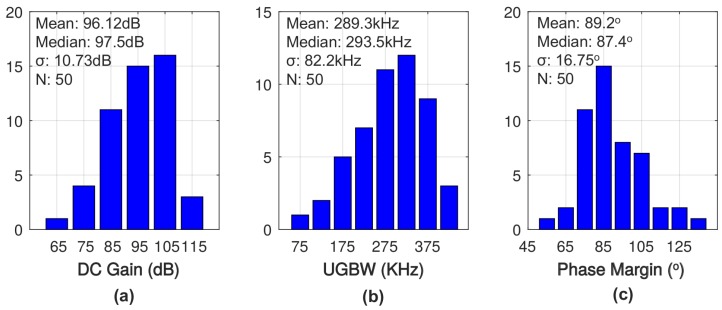
Monte Carlo simulation for (**a**) DC voltage gain (**b**) unity gain bandwidth and (**c**) phase margin of the proposed amplifier with 1-nF load capacitance.

Monte Carlo simulations with 50 samples for DC gain, unity gain bandwidth and phase margin with 1-nF load capacitance are shown in Figure 20. The median performance of 97.5-dB gain, 293.5-kHz unity gain frequency, and 87.4${}^{\circ }$ phase margin is obtained with a standard deviation of 10.7 dB, 82.2 kHz, and 16.8${}^{\circ }$. Considering the limited number of samples, the median performance of the Monte Carlo simulation well matches the simulation results with typical case model. Figure 20c shows that the prototype remains stable with 2σ variation. In addition, the corner simulation performed with one σ process variation presents 110.3-dB gain, 329.9-kHz unity gain bandwidth and 82${}^{\circ }$ phase margin at high side and 95.5-dB gain, 227.3-kHz unity gain band width and 93${}^{\circ }$ phase margin at low side, which also matches the statistical distributions from the Monte Carlo simulation.

**Figure 21:**
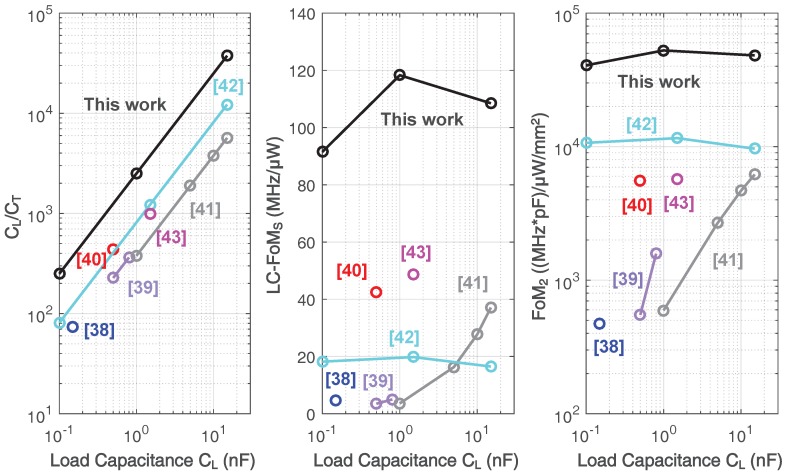
Benchmark of $C_{L}/C_{T}$, LC-${\mathrm{FoM}}_{s}$ and ${\mathrm{FoM}}_{2}$ [38,39,40,41,42,43].

Table 6 compares the simulated performance of the prototype design with the state-of-the-art. Compared to previous works, this work utilizes the smallest compensation capacitance, which is 400 fF, and demonstrates the highest ratio between load capacitance and total compensation capacitance, which is 37,500. In addition, it also presents the best reported performance trade-off on the unity gain bandwidth, load capacitance, and power consumption for a given chip area, as shown in Figure 21.

## 6. Conclusions

Transducers with nonlinear capacitive input impedance need an operational amplifier, which is stable for a wide range of load capacitance. Such an operational amplifier in a conventional design requires a large area for compensation capacitors, increasing costs and limiting applications. In order to address this problem, we present a gain-boosted two-stage operational amplifier, whose frequency response compensation is less sensitive to the load capacitance compared to the conventional Miller frequency compensation. The proposed amplifier cancels the output node pole of the main amplifier stage by the zero of the gain booster, so that a sufficient phase margin can be achieved without using a large compensation capacitor. A prototype CMOS amplifier designed with the proposed architecture uses two-to-four orders of magnitude smaller compensation capacitor compared to the load capacitance. This advantage in the compensation capacitor area requirement extends the applications of the proposed operational amplifier beyond the transducer interface circuits and may benefit general analog integrated circuit applications. The prototype CMOS operational amplifier demonstrates the highest performance trade-off to date when considering unity-gain bandwidth, load capacitance, power consumption, and chip area, which can enable an a compact low-cost transducer interface with unprecedentedly high resolution for emerging applications. It should be also noted that the proposed operational amplifier design technique can be used not only with monolithically integrated transducer interface circuits but also with transducer interface circuits assembled by discrete off-the-shelf components.

## Figures and Tables

**Table 1:** Compensation capacitor size comparison between the PSS amplifier and this work.

	PSS Amplifier [42]	This Work
Second Pole	$1/[\left(C_{1}+C_{2}\right)(r_{o1}\left|\right|r_{\mathrm{oa}2}\left|\right|R_{m})]$	$1/[(C_{1}\left|\right|C_{m}+C_{2})(r_{o1}\left|\right|r_{\mathrm{oa}2}\left|\right|R_{m})]$
UGBW	$A_{v}/\left(C_{f}r_{\mathrm{oa}1}\right)$	$A_{v}/[C_{f}r_{\mathrm{oa}1}g_{\mathrm{mb}}(r_{\mathrm{oa}2}\left|\right|R_{m})]$
$C_{f}$	1	≈$1/[g_{\mathrm{mb}}(r_{\mathrm{oa}2}\left|\right|R_{m})]\approx 0.1$

Note: The same second pole location and unity-gain bandwidth (UGBW) are assumed.

**Table 2:** Transistors size of the proposed work.

Device	Size (μm/μm)	Device	Size (μm/μm)	Device	Size (μm/μm)
$M_{1}$, $M_{2}$	1.7/2.5	$M_{3}$, $M_{4}$	6.55/0.17	$M_{5}$, $M_{6}$	4/0.13
$M_{7}$, $M_{8}$	0.13/0.14	$M_{9}$	3.3/0.2	$M_{10}$, $M_{11}$	5/1
$M_{12}$, $M_{13}$	20/1	$M_{14}$, $M_{15}$	7.5/1	$M_{16}$, $M_{17}$	0.2/0.24
$M_{18}$, $M_{19}$	0.15/0.16	$M_{20}$	0.15/2.52	$M_{21}$	0.15/1.35
$M_{22}$, $M_{23}$	5/5	$M_{24}$, $M_{25}$	0.13/26	$M_{26}$, $M_{27}$	0.13/0.38
$M_{28}$, $M_{29}$	0.15/0.3	$M_{30}$, $M_{31}$	0.15/12.05	$M_{32}$	0.15/0.16
$M_{33}$	0.32/0.17	$M_{34}$	0.2/0.2	$M_{35}$	10/5
$M_{36}$, $M_{37}$	0.29/0.13	$M_{38}$	0.7/0.13	$M_{39}$	0.8/0.13

**Table 3:** Design parameters of the proposed work.

**Transconductance**	**Value (**μ**S)**	**Transconductance**	**Value (** μ**S)**
$g_{m1}$	13.1	$g_{m2}$	15.0
$g_{\mathrm{ma}}$	0.094	$g_{\mathrm{mb}}$	13.2
**Capacitance**	**Value (fF)**	**Capacitance**	**Value (fF)**
$C_{m}$	100	$C_{f}$	200
**Resistance**	**Value (MΩ)**	**Resistance**	**Value (MΩ)**
$R_{m}$	20	$R_{f}$	50

**Table 4:** Phase margin over variations on Miller compensation capacitor.

$C_{L}$ (nF)	This Work	70% $C_{f}$	80% $C_{f}$	90% $C_{f}$	110% $C_{f}$	120% $C_{f}$	130% $C_{f}$
0.08	59.5${}^{\circ }$	55.5${}^{\circ }$	57.2${}^{\circ }$	58.8${}^{\circ }$	60.7${}^{\circ }$	62.7${}^{\circ }$	63.0${}^{\circ }$
0.1	65.2${}^{\circ }$	62.1${}^{\circ }$	63.5${}^{\circ }$	64.7${}^{\circ }$	66.2${}^{\circ }$	67.7${}^{\circ }$	67.9${}^{\circ }$
0.2	73.7${}^{\circ }$	71.0${}^{\circ }$	72.2${}^{\circ }$	73.1${}^{\circ }$	74.8${}^{\circ }$	76.0${}^{\circ }$	77.4${}^{\circ }$
0.5	77.7${}^{\circ }$	76.2${}^{\circ }$	76.8${}^{\circ }$	77.4${}^{\circ }$	78.1${}^{\circ }$	78.9${}^{\circ }$	79.1${}^{\circ }$
1	82.9${}^{\circ }$	80.8${}^{\circ }$	81.7${}^{\circ }$	82.5${}^{\circ }$	83.6${}^{\circ }$	84.6${}^{\circ }$	84.9${}^{\circ }$
2.5	90.9${}^{\circ }$	88.4${}^{\circ }$	89.5${}^{\circ }$	90.3${}^{\circ }$	91.7${}^{\circ }$	92.2${}^{\circ }$	92.9${}^{\circ }$
7	80.9${}^{\circ }$	76.3${}^{\circ }$	78.1${}^{\circ }$	79.5${}^{\circ }$	82.0${}^{\circ }$	82.8${}^{\circ }$	83.7${}^{\circ }$
12	70.2${}^{\circ }$	65.2${}^{\circ }$	66.6${}^{\circ }$	68.5${}^{\circ }$	71.5${}^{\circ }$	72.3${}^{\circ }$	73.1${}^{\circ }$
15	65.8${}^{\circ }$	59.1${}^{\circ }$	61.4${}^{\circ }$	63.3${}^{\circ }$	66.9${}^{\circ }$	68.0${}^{\circ }$	69.5${}^{\circ }$
16	63.9${}^{\circ }$	58.8${}^{\circ }$	60.0${}^{\circ }$	61.9${}^{\circ }$	65.5${}^{\circ }$	66.5${}^{\circ }$	68.1${}^{\circ }$

**Table 5:** Gain margin over variations on Miller compensation capacitor.

$C_{L}$ (nF)	This Work	70% $C_{f}$	80% $C_{f}$	90% $C_{f}$	110% $C_{f}$	120% $C_{f}$	130% $C_{f}$
0.08	8.9 dB	5.0 dB	7.1 dB	8.5 dB	10.6 dB	11.4 dB	12.5 dB
0.1	10.9 dB	6.2 dB	8.4 dB	10.1 dB	11.2 dB	12.0 dB	13.8 dB
0.2	11.2 dB	7.1 dB	9.0 dB	10.8 dB	11.9 dB	13.0 dB	14.8 dB
0.5	13.6 dB	9.7 dB	11.0 dB	12.8 dB	14.2 dB	15.4 dB	16.1 dB
1	28.3 dB	25.4 dB	26.6 dB	27.9 dB	29.4 dB	30.5 dB	31.2 dB
2.5	36.2 dB	32.0 dB	33.4 dB	35.6 dB	37.0 dB	37.8 dB	38.9 dB
7	45.2 dB	41.9 dB	43.1 dB	44.5 dB	46.4 dB	47.7 dB	48.2 dB
12	49.6 dB	46.8 dB	47.6 dB	48.9 dB	51.2 dB	52.1 dB	53.0 dB
15	51.3 dB	48.4 dB	49.3 dB	50.7 dB	52.4 dB	53.0 dB	54.1 dB
16	52.0 dB	48.2 dB	49.6 dB	51.3 dB	53.2 dB	54.1 dB	55.9 dB

**Table 6:** Performance summary and Figure-of-Merit (FoM) comparison.

	[38]	[39]	[40]	[41]	[42]	[43]	This work		
Load Capacitance $C_{L}$ (nF)	0.15	0.5	0.5	1	1.5	1.5	0.1	1	15
$C_{L,\max}/C_{L,\min}$	**N/A**	**1.6**	**N/A**	**15**	**150**	**12**	**150**		
Gain (dB)	>100	>100	>100	>100	≈100	>100	103		
Phase Margin ${(}^{\circ })$	58	70	52	83	87	75	65	83	66
Gain Margin (dB)	22	N/A	8	10	35	N/A	11	28	51
UGBW(MHz)	2.85	4	2	1.37	0.12	3.46	2.78	0.36	0.022
Slew Rate (V/μs)	1.03	2.2	0.65	0.59	5.87	1.46	0.78	0.083	0.006
1% Settling Time (μs)	2.25	0.6	1.23	1.28	4.3	0.57	0.82	11.72	91.82
Power (μW)	45	260	20.4	144	7.4	69.6	7.6		
$V_{\mathrm{DD}}$(V)	1.5	2	1.2	2	1.1	1.2	1		
Technology (nm)	350	350	65	350	180	180	130		
Chip Area $\left({\mathrm{mm}}^{2}\right)$	0.02	0.014	0.0088	0.016	0.0021	0.013	0.00096		
Total Compensation Capacitance $C_{T}$ (pF)	2.02	2.20	1.15	2.64	1.23	1.52	0.40		
$C_{L}/C_{T}$	**74**	**227**	**435**	**378**	**1220**	**987**	**250**	**2500**	**37,500**
${\mathrm{FoM}}_{L}$((V/μs·pF)/μW)	3.5	4.2	15.9	4.1	1190	31.5	10.3	10.9	11.8
${\mathrm{FoM}}_{S}$((MHz·pF)/μW)	9.5	7.7	49.0	9.5	24.3	74.5	36.6	47.3	43.4
LC-${\mathrm{FoM}}_{L}$((V/μs)/μW)	1.7	1.9	13.8	1.5	967.5	20.7	25.8	27.3	29.5
LC-${\mathrm{FoM}}_{S}$(MHz/μW)	**4.7**	**3.5**	**42.6**	**3.6**	**19.8**	**48.7**	**91.5**	**118.3**	**108.5**
${\mathrm{FoM}}_{1}$((V/μs·pF)/μW/${\mathrm{mm}}^{2}$)	175	300	1806	256	566,602	2423	11,453	12,110	13,110
${\mathrm{FoM}}_{2}$((MHz·pF)/μW/${\mathrm{mm}}^{2}$)	**475**	**550**	**5568**	**594**	**11,583**	**5730**	**40,667**	**52,555**	**48,221**

${\mathrm{FoM}}_{L}=\left(\mathrm{SlewRate}\cdot C_{L}\right)/\mathrm{Power}$ ${\mathrm{FoM}}_{S}=\left(\mathrm{UGBW}\cdot C_{L}\right)/\mathrm{Power}$ [37]. LC-${\mathrm{FoM}}_{L}=\left(\mathrm{SlewRate}\cdot C_{L}\right)/\left(\mathrm{Power}\cdot C_{T}\right)$ LC-${\mathrm{FoM}}_{S}=\left(\mathrm{UGBW}\cdot C_{L}\right)/\left(\mathrm{Power}\cdot C_{T}\right)$ [41]. ${\mathrm{FoM}}_{1}=\left(\mathrm{SlewRate}\cdot C_{L}\right)/\left(\mathrm{Power}\cdot \mathrm{ChipArea}\right)$ ${\mathrm{FoM}}_{2}$ = $\left(\mathrm{UGBW}\cdot C_{L}\right)/\left(\mathrm{Power}\cdot \mathrm{ChipArea}\right)$ [51].

## Abbreviations

The following abbreviations are used in this manuscript:

AC	Alternating Current
CMOS	Complementary Metal-Oxide-Semiconductor
CMRR	Common-Mode Rejection Ratio
FoM	Figure-of-Merit
DC	Direct Current
ICMR	Input Common-Mode Range
PM	Phase Margin
PSS	Pseudo-Single Stage
UGBW	Unity Gain Bandwidth

## Appendix A

The bias circuit implemented in this work is illustrated in Figure A1 and Table A1. $I_{1}$ is the supply independent biasing current, which is 50 nA when $V_{\mathrm{DD}}$ is 1 V.

**Table A1:** Transistors size of the bias circuit.

Device	Size (μm/μm)	Device	Size (μm/μm)	Device	Size (μm/μm)
$M_{40}$, $M_{42}$	0.13/3.47	$M_{41}$	0.13/12.54	$M_{43}$	0.13/11.82
$M_{44}$	0.13/3.47	$M_{45}$	1.0/0.34	$M_{46}$	0.13/0.71
$M_{47}$, $M_{49}$	0.13/12.54	$M_{48}$	0.13/0.53	$M_{50}$	0.13/3.47
$M_{51}$	0.13/0.41	$M_{52}$	0.13/3.47	$M_{53}$	0.8/0.34
$M_{54}$	0.13/0.53	$M_{55}$	0.13/12.54	$M_{56}$	0.13/0.24
$M_{57}$	0.13/12.54	$M_{58}$	0.13/3.47	$M_{59}$	0.13/0.53
